# How we treat mantle cell lymphoma with cellular therapy in 2025: the European and American perspectives

**DOI:** 10.1038/s41409-025-02599-x

**Published:** 2025-04-14

**Authors:** Peter Dreger, Sairah Ahmed, Ali Bazarbachi, Sascha Dietrich, Timothy S. Fenske, Nilanjan Ghosh, Olivier Hermine, Mehdi Hamadani

**Affiliations:** 1 Department Medicine V, University of Heidelberg, Heidelberg, Germany; 2 Department of Lymphoma and Myeloma, University of Texas MD Anderson Cancer Center, Houston, TX USA; 3 Bone Marrow Transplantation Program, Department of Internal Medicine, American University of Beirut, Beirut, Lebanon; 4 University Hospital Düsseldorf, Department of Hematology, Oncology and Clinical Immunology, Düsseldorf, Germany; 5 Sarah Cannon Transplant and Cellular Therapy Program, Methodist Hospital, San Antonio, TX USA; 6 Atrium Health Levine Cancer Institute, Wake Forest University School of Medicine, Charlotte, NC USA; 7 Department of Adult Hematology, Necker Hospital, Université de Paris-Cité, Assistance Publique des Hôpitaux de Paris, Imagine Institute, INSERM U1183, Paris, France; 8 Division of Hematology & Oncology, Department of Medicine, Medical College of Wisconsin, Milwaukee, WI USA

**Keywords:** B-cell lymphoma, B-cell lymphoma

## Abstract

Cellular therapies have been cornerstones of the treatment of mantle cell lymphoma (MCL) for decades and have helped to improve the outcome of this formerly very unfavourable B-cell lymphoma considerably. Current established roles of cellular therapies include autologous hematopoietic cell transplantation (HCT) as part of first-line therapy, chimeric antigen receptor-engineered T-cells (CART) for relapsed/refractory MCL, and allogeneic HCT for settings in which CARTs have failed or are unavailable. Therapeutic innovations have recently entered the MCL treatment landscape and are moving upstream in treatment algorithms, challenging the existing management principles. The purpose of this paper is to give some guidance regarding how to best use cellular therapies in this increasingly complex environment. Due to differences in CART labels, available non-cellular treatment options, and philosophy between the American and the European health systems, we found it reasonable to contrast the American and European perspectives on defined standard scenarios, which are often overlapping but show discrepancies in some important aspects.

## Introduction/purpose description

Mantle cell lymphoma (MCL), first recognised as a distinct entity over 40 years ago [1], for a long time was considered as the most unfavourable B-cell lymphoma [2]. During the past decades the outlook of MCL has substantially improved with the introduction of early autologous hematopoietic cell transplantation (autoHCT) [3], high-dose ara-C-based chemoimmunotherapy [4], rituximab maintenance [5], and more recently Bruton’s tyrosine kinase inhibitors (BTKi) and CAR T-cell therapies (CARTs), as effective salvage approaches [6], although the curative perspective even with these more modern treatment options remains uncertain.

A sizable minority of patients harbour morphological and/or biological risk factors associated with poor response to chemoimmunotherapy and autoHCT and limited efficacy of BTKi. These cases are considered as high-risk MCL [7] and include blastoid or pleomorphic histology, a high proliferation index (Ki-67 > 30%), and TP53 gene-related abnormalities [8–13]. In addition, progression or relapse of treated MCL within 24 months from diagnosis (POD24) is associated with substantially reduced survival independent of the aforementioned biological risk factors [12, 14, 15].

Cellular therapy was introduced early into the MCL treatment landscape. AutoHCT was first explored in relapsed/refractory patients [16] but was soon found to be most beneficial when used for consolidation of first-line treatment responses [3, 4, 17–19]. Until recently, autoHCT was widely considered to be a cornerstone of standard frontline therapy, at least for younger patients with MCL [20, 21]. Allogeneic HCT (alloHCT) is arguably the only modality with proven curative potential to date and thus was often pursued as a treatment option for younger, fit, chemosensitive patients in the salvage setting [22]. However, with the recent advent of CARTs, the spectrum of cellular treatment options for MCL has widened considerably. The CD19-directed CAR-T products Brexucabtagene autoleucel (Brexu-cel) received FDA and EMA approval for treatment of r/r MCL in 2020, and Lisocabtagene maraleucel (Liso-cel) in 2024 (FDA approval only). Rapidly, CARTs have replaced alloHCT as first standard option in patients who have failed two lines of systemic therapy including BTKi [21]. Thus, until very recently, the accepted roles of cellular therapies in the MCL treatment landscape were as follows: autoHCT as first-line consolidation; CARTs as second- or third-line treatment, depending on the label; and alloHCT as salvage option after CART failure or unavailability.

Recent developments, however, have challenged this framework. These include the movement of BTKi from the salvage to the frontline setting [10, 23, 24], potentially obviating the need for first-line autoHCT [23]; the maturing evidence that CARTs do not bear curative potential for the majority of patients in the salvage setting [25, 26]; and the introduction of novel therapeutic tools such as non-covalent BTKi, and—as yet not approved—venetoclax, obinutuzumab, bispecific antibodies, and BTK degraders [27–32].

In the absence of sound evidence for the implications and possibilities of these new findings for the management of MCL, the purpose of the present paper is to describe our personal approach to applications, challenges, and perspectives of the established (autoHCT, alloHCT, CARTs) and upcoming (bispecific antibodies) cellular therapies within the evolving treatment landscape of MCL. Because of differences in CART labels, available non-cellular treatment options, and philosophy between the American and the European health systems, we provide perspectives on defined standard scenarios for both the USA and Europe. These are often overlapping but show discrepancies in some important aspects.

## Principles, capacities and limitations of cellular therapies in MCL: current evidence

### AutoHCT

The therapeutic principle of autoHCT is enabling escalation of chemotherapy intensity up to myeloablative doses and/or application of myeloablative total body irradiation (TBI), whereas the purpose of stem cell infusion is simply to restore damaged hematopoiesis without a genuine anti-tumour activity. Accordingly, autoHCT deepens responses to chemoimmunotherapy and prolongs disease control but has no proven curative potential in the vast majority of patients [3, 33, 34], despite some efficacy in patients with high-risk MCL except for TP53 aberrations [8, 35–37]. Although some studies suggest better disease control with TBI over BEAM high-dose therapy, available evidence does not support general superiority of TBI [38, 39]. Apart from the typical acute toxicities and the increased risk of secondary malignancies, the toxicity associated with autoHCT is relatively modest with a 5-year non-relapse mortality (NRM) incidence well below 10% [39–41] (Table 1).

**Table 1:** Strengths and weaknesses of different cellular therapies in MCL.

	autoHCT	alloHCT	CART
Tolerability			
Age/comorbidity restrictions	+	+	(+)
Non-relapse mortality risk	(+)	++	+
2nd neoplasm risk	+	(+)	+
Efficacy			
In refractory disease	negligible	weak	yes
In HR disease^a^	negligible	yes	yes
Curative potential	?	yes	?
Costs	low	high	very high

*CART* chimeric antigen receptor-engineered T-cells, *HCT* hematopoietic cell transplantation.

^a^ High-risk disease: blastoid morphology, KI-67 ≥ 30%, and/or TP53 abnormalities

(+), moderately affecting outcome/eligibility; +, significantly affecting outcome/eligibility; strongly affecting outcome

### AlloHCT

In contrast to autoHCT, the main contributor of the efficacy of alloHCT is graft-versus-lymphoma activity (GVL), an unspecific alloreactivity-based immune effect, which is responsible for its curative potential, but also for the typical toxicities related to graft-versus-host disease (GVHD), infections, and endothelial complications [42, 43]. Accordingly, NRM is considerably higher compared to autoHCT and CART therapies with 1-year incidences between 20% and 30% even after reduced intensity conditioning (RIC) transplants [19, 43, 44]. Similar to other indications, the safety/efficacy profile of alloHCT in MCL is most favourable if performed in a chemo-sensitive disease status [19, 43], but it can also be effective in a minority of patients undergoing transplantation with refractory MCL [43, 45], and in patients with high-risk MCL [14, 44, 46]. In the absence of outcome advantages of myeloablative over reduced-intensity conditioning [47–50], RIC has become standard of care in MCL allotransplants, making the procedure accessible also for older and more comorbid patients [51]. Similar to other standard indications, matched unrelated and haplo-identical donors appear to be reasonable alternatives to matched related donors also in MCL [48, 52, 53], meaning that donor unavailability is no longer a bottleneck in MCL allotranplants [44, 54]. The overall risk of second primary cancers of allotransplanted patients appears to be similar to that of the general population except for certain solid neoplasms involving skin, mucous membranes, and bone [55].

### CART

Unlike alloHCT, CAR-T cell therapies are *targeted* cellular immunotherapies. Due to the autologous origin of the CARTs currently available for MCL, there is virtually no GVHD risk if the patient has not undergone a prior alloHCT [56, 57]. However, with the most widely used commercial product (Brexu-cel) typical CART-associated toxicities, in particular neurotoxicities and infections, are relatively pronounced, contributing to a 1-year NRM incidence around 10% [26, 58, 59]. Liso-cel has also been associated with a considerable risk of infections in its pivotal MCL trial, with a crude NRM rate of more than 10% [60]. Despite high overall and complete response rates, even in refractory and high-risk MCL, a continuing pattern of relapse has been observed without clear plateaus of duration of response curves at least with brexu-cel where longer follow-up is already available (Table 2). This implies that the curative potential of CD19 CARTs in MCL remains uncertain [25, 26, 44, 60, 61]. In addition, there appears to be a risk of secondary neoplasms, in particular t-MDS/AML [59], whereas the risk and causal relation of peripheral T-cell lymphomas subsequent to CAR-T therapy is still a matter of debate [62–67].

**Table 2:** Pivotal trials of CARTs labelled for r/r MCL.

	Brexucel (ZUMA-2 cohort 1) [25, 84, 97]	Lisocel (TRANSCEND NHL 001/MCL) [60]
Study type	Phase-2	Phase-2
Eligibility	r/r incl. BTKi; PS 0-1; no CNS disease; no prior alloHCT; ≤5 prior lines	≥2 L incl. BTKi; PS 0-1
*N*		
# apheresed (%)	74	104
# Treated set (%)	68 (92%)	83 (80%)
Period	2016–2019	2016–2022
Age (years; median (range))	65 (38–79)	69 (36–86)
PS > 0 (ECOG)	n.a.	45%
High-risk features		
Ki-67 ≥ 30%	82%	75%
Blastoid/pleomorph.	31%	31%
TP53abn	17%	23%
POD24	49%	n.a.
2nd CNS involvement	-	8%
Prior lines/median (range)	3 (1-5)	3 (1-11)
Prior autoHCT	43%	30%
Prior alloHCT	-	7%
BTKi refractory	62%	53%
Holding/Bridging	BTKi/steroids	Any
Received Bridging	37%	66%
BTKi	76%	n.a.
Response to Bridging	low	n.a.
Toxicity		
Neurotox ≥ G3	31%	9%
Late ICAHT ≥ G2	≥16%	24%
Severe infection	32%	15%
ICU admission	n.a.	7%
Non-relapse mortality	7.4%^b^	18%^a^
Best ORR/CR	92%/68%	87%/75%
PFS from infusion (12mo)(median)	62%/25mo	53%/15mo
OS from infusion (12mo)	81%/47mo	62%/18mo
Median follow-up (months)	68	24

*BTKi* Bruton’s tyrosine kinase inhibitors, *HCT* hematopoietic cell transplantation, *ICAHT* immune effector cell associated hematotoxicity, *ICU* intensive care unit, *ORR* overall response rate, *OS* overall survival, *PFS* progression-free survival, *POD24* progression of disease within 24 months, *PS* performance status, *r/r* relapsed/refractory.

^a^Crude rate.

^b^Point estimate according to Cordas Santos et al. [59].

### Bispecific antibodies

Similar to CARTs, bispecific antibodies are targeted immunotherapies directing T-cells to tumour cells in vivo independent of their T-cell receptor specificity, for B-cell lymphoma mostly using anti-CD20/anti-CD3 fusion molecules. Thus, they act as cellular immunotherapy inducers rather than being cellular therapies by themselves, which has the advantage that the medicinal product is a protein and not a cell, making ex vivo procurement and associated efforts and time delays unnecessary. Although infections remain a significant threat, the risks of severe CRS and neurotoxicity appear to be 5–10 fold lower than that observed with CARTs [30, 68]. Regarding MCL, the published experience with bispecific antibodies in MCL is limited, and to date there is still no approval for this indication. However, preliminary data suggest that response rates could be of a similar magnitude as reported for Brexu-cel and Liso-cel [30], although the durability of these responses is still unknown with relatively short follow-up available.

## 1L cellular therapy indications

### AutoHCT

Based on pioneering work by American and European researchers [16, 17, 69–71], high-dose ara-C-based chemoimmunotherapy with autoHCT consolidation has been considered a standard first-line therapy in younger patients (≤65 years) since the 2000s (GLSG, Nordic and LYSA trials) [3, 4, 18, 21, 35]. With this approach, 4-year PFS rates of ≥70% can be achieved, and ≥80% if 3 years of rituximab maintenance is added [5, 72]. However, a recent interim analysis of the randomised U.S. NCTN trial EA4151 comparing consolidative autoHCT vs no autoHCT in patients with treatment-naïve MCL achieving both metabolic CR and MRD clearance (at the 1 × 10^−6^ sensitivity level) (Clonoseq®) after rituximab-based chemoimmunotherapy could not prove a benefit of 1st-line autoHCT in MRD-negative patients [73]. There was a suggestion of benefit to autoHCT in the patients who remained MRD-positive after induction [73]. In addition, recent trials exploring BTKi as part of first-line treatment of MCL question the role of upfront high-dose therapy [23, 24]. In particular, the large TRIANGLE trial compared standard frontline therapy (ara-c-based chemoimmunotherapy followed by autoHCT consolidation and rituximab maintenance) without (A) or with ibrutinib (A + I) with a third arm with ibrutinib but without autoHCT (I). This trial did not show a benefit of autoHCT consolidation in terms of failure-free survival when ibrutinib is given as part of induction and maintenance therapy Dreyling, 2024 11648 /id}. However, it has to be kept in mind that the follow-up of TRIANGLE is still too short to exclude inferiority of I vs A + I beyond the 4-year landmark after rituximab maintenance has ended [74]. In addition, subgroup analyses from the TRIANGLE trial showed trends toward failure-free survival benefit for autoHCT in certain high-risk subsets, despite receiving ibrutinib with induction and maintenance. These include patients with proliferation rate >30%, blastoid histology, and increased p53 expression [75]. It also remains unclear whether replacement of rituximab with obinutuzumab in arm A would provide additional benefit and potentially obviate the need for ibrutinib or autoHCT in the first-line setting [72]. Another relevant question is whether newer BTKi (acalabrutinib, zanubrutinib, pirtobrutinib), would provide similar benefit but less toxicity compared to ibrutinib in this first-line setting [24].

First-line autoHCT in MCL is also challenged by recent phase 2 data suggesting that the combination of the BTKi zanubrutinib, the anti-CD20 antibody obinutuzumab, and the bcl-2 inhibitor venetoclax (the ‘BOVen’ regimen) administered for 24 cycles can provide a 2-year PFS of more than 70% in treatment-naïve patients with TP53-mutated MCL [76] These observations challenge the results seen in the TRIANGLE I and A + I arms [75], but clearly longer follow-up is needed to confirm the efficacy of this regimen. Although none of the BOVen components is currently approved for first-line treatment of MCL, in the U.S. the BOVen regimen is listed as a suitable option for TP53-mutated patients in the NCCN guidelines [77].

### CART

CARTs as part of first-line therapy in MCL have to be considered as experimental and should be performed only within clinical trials [21], such as NCT05495464 and NCT064822684, both addressing the impact of Brexu-cel therapy in a BTKi- and rituximab-containing framework in treatment-naïve patients with high-risk MCL.

### AlloHCT

Given its risks on the one side and the excellent outlook with modern first-line therapies on the other side, upfront alloHCT has no established role in today’s MCL treatment algorithms, even within clinical trials [19, 21, 78].

Our first-line treatment recommendations based on these considerations are summarised in Table 3.

**Table 3:** How we treat with cellular therapy in 1 L.

	The European perspective	The American perspective
Standard risk MCL, BTKi available	Follow TRIANGLE arm A + I or I (=no autoHCT) after discussion of the uncertainties associated with the lack of long-term follow-up in TRIANGLE	Follow TRIANGLE arm I (using Acalabrutinib or Zanubrutinib), or use a rituximab/BTKi combo
Standard risk MCL, BTKi not available for 1 L therapy	Follow TRIANGLE arm A (with obinutuzumab maintenance if available)	Follow EA 4151^a^ if PET and MRD-negative to 1 × 10^−6^ sensitivity (=3 years rituximab maintenance but no autoHCT). Consider autoHCT in MRD-positive chemosensitive cases.
High risk MCL^b^, BTKi available	Treat on clinical CART trial if available, otherwise follow TRIANGLE arm A + I (or I if PET/MRD-negative after induction)	Treat on clinical trial if available. Otherwise, treat non-TP53-mutated as standard risk above or use a rituximab/BTKi combo. Avoid bendamustine regimens in CART eligible patients For TP53 mutated, use BOVen regimen
High risk MCL^b^, BTKi not available for 1 L therapy	Treat on clinical CART trial if available, otherwise follow TRIANGLE arm A	Scenario unlikely since acalabrutinib is approved for 1 L and NCCN guidelines support use of second-generation BTKi in 1 L

^a^EA4151 allowed various induction regimens—we recommend either Nordic MCL-2 (maxi-RCHOP alternating with R-araC for 6 cycles)); RCHOP alternating with RDHAP (6 cycles); DHAX (dexamethasone, high-dose araC, oxaliplatin for 4 cycles); BR/CR (Bendamustine/ rituximab, cytarabine/rituximab for 6 cycles); or BR = bendamustine/ rituximab (6 cycles).

^b^Defined as: proliferation rate >30% or blastoid variant, over-expression of P53 by immunohistochemistry, or TP53 mutated.

## 2 L cellular therapy indications

### AutoHCT

In the pre-rituximab/BTKi era, salvage autoHCT resulted in 4-year PFS rates of 30–60% if administered to HCT-naïve patients with chemosensitive MCL [19]. Because the first-line treatment standard contained autoHCT until recently, nowadays there are few transplant-eligible patients arriving HCT-naïve in the salvage setting [12, 14]. This situation may change if 1 L autoHCT will be increasingly replaced by BTKi. However, the majority of early relapses on BTKi-based 1 L regimens will be characterised by high-risk features, and the feasibility and efficacy of autologous transplantation in BTKi-refractory patients is largely unclear. There will be other 2 L patients who are BTKi-naïve, or who have been off BTKi therapy for many years. For such patients, BTKi will be a logical 2 L option. In addition, CART is an available option (at least in the U.S.) for 2 L patients. As a result, a renaissance of salvage autoHCT for r/r MCL patients in 2 L seems unlikely in the coming years, contributing to recent trends of decreasing use of autoHCT in lymphoma [79].

### CART

While in Europe Brexu-cel is approved only after two systemic lines including a BTKi, the FDA label states only ‘relapsed or refractory MCL’. In contrast, Liso-cel has an FDA label similar to the EMA Brexu-cel label, and is not yet approved for MCL in Europe. This implies that Brexu-cel can be used in the US on label as 2 L therapy in BTKi-naïve patients although the evidence supporting its use in this setting is still limited. ZUMA-2 cohort 3 explored Brexu-cel in 95 patients with BTKi-naïve r/r MCL, the majority of them in 2 L, and almost half of the patients evaluated harboured high-risk features such as high KI-67 and/or TP53 abnormalities. Despite a high CR rate of 73%, the median PFS was 27 months and thus comparable to that reported for the BTKi-exposed cohort 1 (26 months) [25, 80]. Similar results were suggested by a real-world analysis comparing 24 BTKi-naïve patients with 144 BTKi-exposed patients with largely high-risk r/r MCL [26]. Thus, 2 L Brexu-cel is not clearly superior to 2 L ibrutinib monotherapy in r/r MCL, at least in the absence of high-risk criteria [9]. High-risk/POD24 patients put on 2 L BTKi should be monitored closely and CART considered early in case of insufficient response [81].

In the absence of reasonable therapeutic alternatives, patients who have progressed on 1 L BTKi appear to be eligible for 2 L CART treatment if reimbursement can be assured or a clinical trial is available.

### AlloHCT

Durable disease control with 3-year PFS rates around 60% has been reported for patients with chemosensitive r/r MCL and TP53 abnormalities and POD24, respectively, who had undergone consolidative alloHCT [14, 46]. In places where CARTs are not available alloHCT could be considered as a second-line consolidation option in patients with high-risk MCL and/or inadequate response to BTKi [21] (Table 4).

**Table 4:** How we treat with cellular therapy in 2 L.

	The European perspective	The American perspective
BTKi-naïve + autoHCT-naïve; no high-risk features	Consider autoHCT if CR on salvage chemotherapy (non-BTKi)	Consider autoHCT in young patients with long first remission, who are now in 2^nd^ CR on a case-by-case basis. Otherwise favour BTKi
BTKi-naïve, autoHCT-exposed; no high-risk features	BTKi without cellular therapy consolidation if CR is achieved	
BTKi-naïve + high-risk features	BTKi but consider CARTs (or alloHCT in case of CART unavailability) early in case of insufficient response; or CART trial rightaway.	BTKi followed by CART consolidation. In older/unfit patients responding to BTKi, CARTs may be reserved for the next line
BTKi-exposed	**CART**. Consider BTK re-treatment if several years since BTKi exposure and patient did not progress on BTKi previously	

## >2L cellular therapy indications

### AutoHCT

In the absence of evidence for BTKi/CART-exposed patients and due to caveats outlined previously (see 2L section above) autoHCT will be an option only in rare chemosensitive cases beyond the second line.

### CART

Because virtually all patients will have received a BTKi during first or second line, CART appears to be the treatment of choice in all eligible CART-naïve patients in the 3+ line setting. The two approval trials (ZUMA-2 for Brexu-cel and TRANSCEND NHL001 for Liso-cel) as well as a number of larger real-world studies (all with Brexu-cel) concordantly show high CR rates of 65–80% in this otherwise very dismal setting, translating into prolonged disease control with 12-month PFS rates between 50% and 70% (Tables 2 and 5). This accounts also for patients with high-risk MCL including POD24, even though in some studies the outcome of these subsets tended to be worse [26, 60, 82, 83].

**Table 5:** Brexucel real-world studies in r/r MCL.

	UK [82]	DESCAR-T [87]	SIE [83]	DRST/GLA/ SAKK [98]	US CART consortium [26]
Study type	Registry retrospective	Registry retrospective	Registry retrospective	Registry retrospective	Registry retrospective
Eligibility	≥2L incl. BTKi; PS 0-1; no CNS disease	EMA label	≥2L; r/r to BTKi; ZUMA-2 eligible	EMA label	FDA label
*N*					
# intended	119	178	n.a.	n.a.	n.a.
# apheresed (%)	104 (87%)	n.a.	n.a.	n.a.	189
# infused (%)	83 (70%)	152 (85%)	106	111	168 (89%)
Period	2021–2023	2019–2023	2019–2024	2021–2023	2020–2021
Age (years; median (range))	68 (41–80)	68 (39–83)	63 (42–79)	68 (50–84)	67 (34–89)
PS > 1 (ECOG)	0	12%	0	7%	14%
High-risk features					
Ki-67 ≥ 30%	78%	79%	54%	79%	78%
Blastoid/pleomorph.	42%	31%	30%	33%	40%
TP53abn	53%	30%	29%	27%	48%
POD24	57%	n.a.	42%	n.a.	51%
Prior lines/median (range)	2 (2–7)	3 (1–9)	3 (2–5)	3 (1–8)	3 (1–10)
Prior autoHCT	34%	40%	58%	55%	28%
Prior alloHCT	13%	6%	-	8%	3%
BTKi refractory	30%	n.a.	35%	58%	76%
Holding/Bridging	90%	83%	79%	74%	68%
CIT	50%	n.a.	31%	35%	34%
BTKi ± CD20.	17%	n.a.	45%	28%	24%
Venetoclax ± x	10%	n.a.	13%	20%	27%
Response to Bridging	41% (37/91)	41% (51/125)	18% (15/83)	65% (42/65)	33% (32/95)
Toxicity					
Neurotox ≥ G3 (any)	22% (55%)	15% (55%)	18% (48%)	28% (54%)	32% (61%)
Late ICAHT ≥ G2	59%	9% (≥G3)	4.4% (≥G3)	n.a.	>18%
Severe infection	>30%	>25%	n.a.	31%	>21%
ICU admission	27%	34%	18%	22%	20%
T cell recovery 6mo	na	na	na	23%	na
Non-relapse mortality (12mo)	18%^d^	18%^a^	7.3%	10%^a^	7.1% 10.7%^d^
Best ORR/CR	87%/81%	85%/72%	88%/75%	88%/64%	90%/82%
PFS from infusion (12mo)	62%	46%	62%	69%	69%
OS from infusion (12mo)	74%	70%^c^	82%	74%	82%
Median follow-up (months)	13	12	12	12	14

*BTKi* Bruton’s tyrosine kinase inhibitors, *CIT* chemoimmunotherapy, *EMA* European Medical Agency, *FDA* Federal Drug Administration, *HCT* hematopoietic cell transplantation, *ORR* overall response rate, *OS* overall survival, *PFS* progression-free survival, *PS* performance status, *r/r* relapsed/refractory,

^a^Crude rate.

^b^Relapsed and primary refractory patients, respectively.

^c^Estimated from published survival plot.

^d^Point estimate according to Cordas Santos et al. [59].

However, even with longer follow-up in the approval trials no plateau in the survival curves has become evident, with median durations of PFS and OS between 15 and 25 months. Another concern is the relatively high NRM rate (7–18% at 12 months), with infections being the most important contributor to NRM, even though it has to be taken into account that TRANSCEND NHL001 as well as all real-world studies were adversely affected by the COVID pandemic. In addition, Brexu-cel is associated with a considerable risk of severe neurotoxicity (≥G3 in 15–30%), which appears to be a particular burden in the mostly elderly MCL population. A labelled alternative in this setting, in particular in patients with comorbidities putting them at higher NRM risk with CART, is the non-covalent BTKi pirtobrutinib which has an excellent safety profile but clearly inferior efficacy with a CR rate of 20% and a median PFS < 8 months [27].

### CART eligibility

Although age ≥65 appears to be associated with an increased NRM [82], similar to LBCL a clear-cut age limit for MCL CARTs cannot be defined [60, 82, 84]. Regarding the impact of comorbidities, informative MCL-specific analyses are not available, but it appears to be reasonable to follow the same policies as in LBCL CARTs, suggesting that, rather than a single criterion, PS, age, and comorbidities collectively form CART eligibility [85]. There is no reason in terms of safety and efficacy to withhold CARs from patients with MCL CNS involvement [26, 86].

### Holding/bridging prior to CART

Holding therapies are defined as those administered between indication for CART and leukapheresis; and bridging therapies as those delivered between leukapheresis and CART infusion [85]. While a survival benefit for patients in whom bridging was considered not necessary or who responded to bridging has been observed in the French Brexu-cel real-world study [87], similar effects of bridging therapy were not found in the US and UK analyses [26, 82], nor in the approval trials [60, 84]. Moreover, there is a paucity of evidence showing a correlation between pre-lymphodepletion tumour burden and tumour activity and CART outcome in MCL. Thus, similar to LBCL, the practical value of holding/bridging strategies remains unsettled, but there are some theoretical aspects arguing in favour of bridging also in MCL, such as symptom control, patient stabilisation during the pre-CART phases, and mitigating pro-inflammatory effects of uncontrolled lymphoma [85].

Suggested holding/bridging regimens in covalent BTKi-refractory patients include pirtobrutinib as an on label option [27], and combinations of venetoclax with covalent BTKi and CD20 antibodies [28, 32]. Another, still off-label bridging option is bispecific antibodies [30]. Conventional chemoimmunotherapy can be attempted for holding/bridging with the exception of bendamustine-containing regimens because of their severe lymphodepleting effects [25, 26, 88].

### AlloHCT

Although there are numerous studies demonstrating the efficacy of alloHCT in MCL, there is only limited evidence for its feasibility in BTKi-pretreated patients (Table 6), and virtually no informative data on alloHCT post-CD19 CART in MCL. The EBMT recently published a study on a dataset of 272 patients who underwent alloHCT for MCL after BTKi exposure. With 12-month estimates for OS, PFS, NRM, and relapse incidence of 66%, 56%, 19%, and 26%, respectively, OS and NRM, but not PFS and relapse incidence, were significantly inferior to the ZUMA-2 patient population on multivariate Cox regression analysis. Similarly, OS and NRM, but not PFS and relapse incidence, showed significant benefit for the ZUMA-2 population on propensity score matching with an equal number of alloHCT recipients using age, gender, pretreatment, and time from diagnosis as matching factors [44] (Table 6). Altogether, this study supports the policy of preferring CART over alloHCT as first cellular immunotherapy in BTKi-exposed patients with r/r MCL because of the NRM-related survival advantage, even if disease control may be better with alloHCT in the long-term.

**Table 6:** AlloHCT for r/r MCL in patient exposed to BTKi: retrospective studies.

	EBMT 2019 [99]	UK/Italy R-BAC [100]	EBMT 2025 [44]
*N*	22	11	64
Period	2013–2016	2015–2019	2014–2020
Age (years; median (range))	59 (45-69)	62 (43-67)	>60
PS > 1 (ECOG)	0	n.a.	n.a.
High-risk features^a^			
Ki-67 ≥ 30%	n.a.	n.a.	65%
Blastoid/pleomorph.	n.a.	n.a.	54%
Prior lines/median (range)	2 (1–5)	n.a.	3 (1–5)
Prior autoHCT	86%	n.a.	44%
BTKi refractory	18–27%	100%	n.a.
Donor		n.a.	
MRD	27%		27%
Unrelated	73%		49%
Haplo	-		23%
Cord blood	-		2%
Conditioning		n.a.	
Reduced	73%		91%
Myeloablative	27%		9%
TBI-based	32%		31%
Non-relapse mortality (12mo)	5%	0	21%
Relapse (12mo)	19%	0	28%
PFS (12mo/24mo)	76%/76%	100%/80%	50%/44%^b^
OS (12mo/24mo)	86%/86%	100%	59%/51%^b^
Median follow-up (months)	13 (2–29)	10	34 (27–50)

*BTKi* Bruton’s tyrosine kinase inhibitor, *HCT* hematopoietic cell transplantation, *MRD* matched related donor, *OS* overall survival, *PFS* progression-free survival, *PS* performance status, *TBI* total body irradiation.

^a^At diagnosis.

^b^Estimated from published survival plot.

Accordingly, alloHCT remains a standard option in eligible salvage-responsive patients after CART failure, or who progress on BTKi and have no access to CART. It might be also considered as consolidation in the minority of patients not achieving CR on CART [21]. This would require pre-emptive donor search at least in those patients who have an increased risk of CART resistance (i.e. high-risk MCL). For response induction before alloHCT, the same considerations account as for holding/bridging prior to CART. Whether highly sensitive MRD might be useful to identify patients destined to relapse after CART, and who therefore would benefit from ‘pre-emptive’ alloHCT prior to overt relapse, remains to be determined.

### AlloHCT eligibility

With the upper age limit for alloHCT gradually increasing [51, 89], patient selection for alloHCT should be based on individually weighing PS, age, and comorbidities similar to the approach described for CART, albeit much more cautiously.

Our >2 L treatment recommendations based on these considerations are depicted in Table 7.

**Table 7:** How we treat with cellular therapy in >2L.

	The European perspective	The American perspective
CART-naïve	**CART;** consider BTKi-venetoclax combination trials or pirtobrutinib as alternative in standard-risk MCL, in particular in patients with comorbidities putting them at higher NRM risk with CART. Consider alloHCT in eligible patients in case of CART unavailability. In any case start donor search in eligible patients >2L	
r/r after CART	Consider alloHCT in eligible patients who respond to salvage therapy	
PR as best response 3 months after CART	Consider consolidative alloHCT in eligible patients, potentially after trying to deepen response	

### Bispecific antibodies

In a phase I/II trial, glofitamab achieved a CR rate of 71% in 31 patients previously treated with a BTKi, with a median duration of CR of 13 months [30]. Similar response rates were observed with the combination of the bispecific antibody mosunetuzumab and the antibody drug compound polatuzumab vedotin in another trial including 20 patients with r/r MCL having failed BTKi [90]. Compared to CART, the safety profile in both trials was relatively favourable. More evidence and longer follow-up with these and other bispecific antibodies are needed to assess whether this drug class can be an alternative to CART as cellular immunotherapy beyond the second line.

## Future challenges and perspectives

With the increasing speed of cellular and non-cellular therapeutic innovations entering the MCL treatment landscape, and with already established modern MCL therapies moving upstream the management algorithms, it is clear that the suggestions given in this paper can only be a snapshot. Novel candidate agents or concepts holding promise for further improving MCL treatment options include alternative BTK-tackling agents, CARTs and bispecific antibodies addressing targets other than CD19 and CD20 [31, 91], and, in addition, strategies aimed at augmenting CART efficacy, and of course the plethora of possible combinations of CARTs, bispecific antibodies and molecular therapies with each other, just to name e few [92–96]. This implies that in the future it will become even more important to continuously rearrange the increasingly complex MCL management toolkit based on evidence and rational considerations in order to find the best path out of MCL for our patients.
